# GPU-Based Parallel Processing Techniques for Enhanced Brain Magnetic Resonance Imaging Analysis: A Review of Recent Advances †

**DOI:** 10.3390/s24051591

**Published:** 2024-02-29

**Authors:** Ayca Kirimtat, Ondrej Krejcar

**Affiliations:** Center for Basic and Applied Research, Faculty of Informatics and Management, University of Hradec Kralove, Rokitanskeho 62, 500 03 Hradec Kralove, Czech Republic; ondrej.krejcar@uhk.cz

**Keywords:** parallel processing, GPU, MRI, review

## Abstract

The approach of using more than one processor to compute in order to overcome the complexity of different medical imaging methods that make up an overall job is known as GPU (graphic processing unit)-based parallel processing. It is extremely important for several medical imaging techniques such as image classification, object detection, image segmentation, registration, and content-based image retrieval, since the GPU-based parallel processing approach allows for time-efficient computation by a software, allowing multiple computations to be completed at once. On the other hand, a non-invasive imaging technology that may depict the shape of an anatomy and the biological advancements of the human body is known as magnetic resonance imaging (MRI). Implementing GPU-based parallel processing approaches in brain MRI analysis with medical imaging techniques might be helpful in achieving immediate and timely image capture. Therefore, this extended review (the extension of the IWBBIO2023 conference paper) offers a thorough overview of the literature with an emphasis on the expanding use of GPU-based parallel processing methods for the medical analysis of brain MRIs with the imaging techniques mentioned above, given the need for quicker computation to acquire early and real-time feedback in medicine. Between 2019 and 2023, we examined the articles in the literature matrix that include the tasks, techniques, MRI sequences, and processing results. As a result, the methods discussed in this review demonstrate the advancements achieved until now in minimizing computing runtime as well as the obstacles and problems still to be solved in the future.

## 1. Introduction

Real-time (digital) and post-examination (analog) image processing are the two types of methodologies utilized in image processing. Printouts and photos in hard copy can be processed using analog image processing. Image analysts employ these visual approaches in conjunction with a variety of interpretive foundations. Real-time processing is much more challenging in medical image segmentation because there are a lot of data in an image that needs to be processed. Moreover, according to [1], interactive, real-time processing and rendering of data on immersive, high-resolution screens is a difficult task that necessitates the development of sophisticated techniques for computer graphics, data management, and parallelization, and it is projected that future datasets in this subject would grow to TB scale. Therefore, developments in parallel processing algorithms utilizing graphic processing units (GPUs) [2,3,4,5] or multi-core processors have advanced, and these algorithms are greatly accelerating the segmentation process. In fact, with the initiation of GPUs, powerful, affordable, and versatile parallel processing platforms were used by numerous successful studies in the domains of intelligent computation and image processing [6]. Furthermore, the medical community has already recognized the advantages of parallel processing algorithms because of real-world experiments carried out in multiple hospitals [7]. On the other hand, the traditional computational Central Processing Unit (CPU) cannot process medical image data quickly enough due to the abrupt increase in data sizes. Rather, the GPU began to emerge as a cutting-edge technology for solving challenging computational issues in the field of medicine [8].

**Figure 1:**
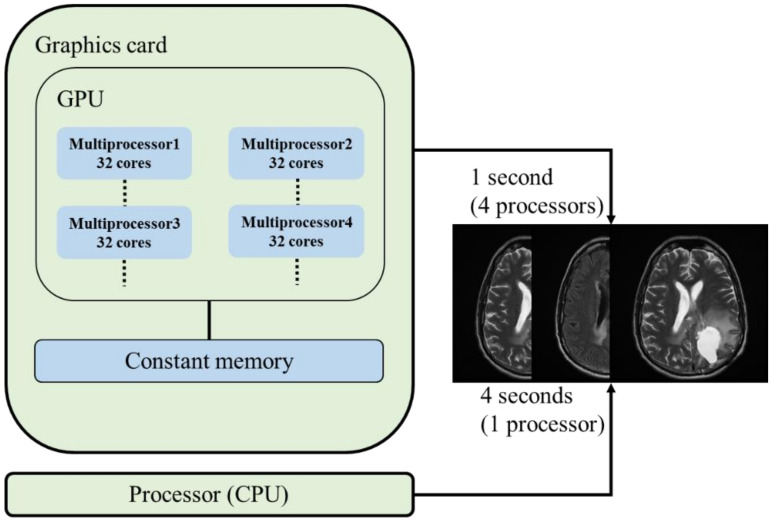
Typical CPU and GPU for brain MRI processing.

Generally speaking, the GPU is a trendy phrase with a complex architecture that differs across models, and since GPU computing is very inexpensive and energy-efficient, it can also be used to speed up parallel processing. Truthfully, the biggest motivation behind GPU adoption is its increased power for realistic computer tasks. Although CPUs had the best computational performance in the past, GPUs now have higher computational performance than CPUs, as seen in Figure 1. In addition, advanced computer graphics are needed for computer rendering in order to generate complicated scenarios in less than 25 ms. Moreover, the rendering process is carried out in parallel on individual pixels by taking advantage of an image’s Single Instruction Multiple Data (SIMD), a form of data-level parallelism. Also, a highly appropriate SIMD algorithm that operates on thousands of threads concurrently is executed by today’s GPUs [9].

On the other hand, the most frequently used medical imaging modality for brain imaging is magnetic resonance imaging (MRI), followed by computed tomography (CT), positron emission tomography (PET), and ultrasound [10,11,12,13]. In basic terms, MRI has been widely utilized to analyze the anatomy of the entire brain [14]. The brain, spinal cord, and vascular architecture can all be seen in high detail on an MRI machine because of its strong contrast. Moreover, this method is safer than CT because it does not use radiation [15]. The three most often utilized MRI sequences are FLuid Attenuated Inversion Recovery (FLAIR), T1-weighted, and T2-weighted [16,17]. MRIs are typically performed using sophisticated, high-priced equipment, and computers as part of a medical evaluation process aimed at determining the best course of treatment for brain disorders. Furthermore, MRI data acquisition and image rendering are time-consuming processes, and for this reason, accurate and quick brain MRI processing are required in order to expedite the process [18,19,20]. Also, because of certain artifacts in brain MRI images, image segmentation is thought to be one of the most challenging tasks when processing these images. Three purposes are served by GPU-based medical image segmentation: first, it allows for the comparison of several candidate image segmentation algorithms; second, it makes the segmentation of large datasets easy and automated; and third, it enables interactive GPU segmentation and visualization. These three combined goals facilitate the rendering of GPU memories at very high speeds [9].

Furthermore, medical image segmentation [21], which is very popular among scientists [22], is frequently used to separate tumors, bones, organs, and brain structures. When it comes to segmenting medical images [23], thresholding, clustering, level sets, active contours, region-growing, and other algorithms are the most helpful. Image segmentation is one of the trickiest parts of processing brain MRI images because various artifacts exist in the acquired images. Nevertheless, there is no single, all-encompassing technique to effectively get around the computational burden of image segmentation due to the variations in MRI images. Three objectives are served by GPU-based medical image segmentation: first, to compare various candidate image segmentation algorithms; second, to quickly and automatically segment large datasets; and third, to offer interactive GPU segmentation and visualization [9]. Given that modern GPUs have hundreds of cores and can simultaneously employ thousands of threads, parallel programming models like CUDA have the potential to significantly improve performance. As a result, GPUs have been heavily utilized in recent years to solve challenging computational problems.

For both CPU and GPU, there are evaluation metrics such as memory access and utilization, temperature and power usage, training time, and accuracy. For instance, training a GPU on a larger batch size uses a significant amount of memory in comparison to training on a smaller batch size. However, CPU memory consumption is unaffected by changes to hyperparameter values. In addition, training time differs greatly when compared between GPU and CPU. We can see that this difference is evident in the study by [24], which conducted an experiment training a dog–cat classifier for 20 epochs on both CPU and GPU. Based on the results, the total training time for the CPU was around 13 h for 20 epochs, yet it took only around 2 h to train on the GPU with a batch size of 64. The test accuracy seems to be 99% for both CPU and GPU.

In this extended review, we investigate GPU-based parallel processing methods, expanding on a thorough search of the Web of Science (WoS) literature between 2019 and 2023 for the analysis of brain MRIs. In the area of brain MRI computation by parallel processing, we also cover a variety of implementations, solutions, and parallel approaches. This review is the extended version of our earlier conference paper [25], presented in the IWBBIO2023 conference. Numerous experiments with different parameter settings and algorithms are also possible because GPU-based parallel processing methods can be used to solve a wide range of computation problems in the field of brain MRIs. Furthermore, in terms of frequency and FLOPs (floating-point operations per second), a single GPU core is generally slower than a CPU core. Nevertheless, in terms of parallelized performance, GPU outperforms CPU due to its larger core count. In addition to significant advancements in the field that make high-level programming interfaces possible, this review aims to provide insightful commentary and a gentle introduction to readers who are keen to learn about the specifics of GPU programming, parallel processing, and brain MRI analysis.

In the continuation of this review study, we introduce medical imaging techniques and discuss them separately in subsections of Section 2, whereas the procedure and the results of this literature review are provided in the subsections of Section 3. We present our review findings in Section 4. In Section 4, we also carry out a discussion based on GPU-based parallel processing implementations and prominent case studies. The future trends, prospects, and concluding remarks are provided in Section 5 of this review paper.

## 2. Medical Imaging Techniques

From the moment it became feasible to scan and import medical images onto a computer, scientists have developed automated analysis tools. Medical image analysis was first performed using a sequential application of low-level pixel processing (such as region growing and edge and line detector filters) and mathematical modeling (such as fitting circles, ellipses, and lines) to create complex rule-based systems that addressed specific tasks between the 1970s and 1990s. As a result, we have witnessed a transition from fully human-designed systems to those that are computer-trained, utilizing sample data from which feature vectors are derived.

Therefore, in the subsections of this section, we introduce different medical imaging methods used in the existing literature, such as image classification, object detection, image segmentation, registration, and content-based image retrieval.

### 2.1. Image or Exam Classification

One of the first applications of deep learning-based parallel processing approaches in medical image analysis was the categorization of images or exams. Exam classification usually involves one or more images (an exam) as input and one diagnostic variable (for instance, whether the illness is present or not) as the output. Every diagnostic test is a sample in this context, and dataset sizes are usually smaller than those in computer vision (for instance, hundreds/thousands vs. millions of samples) [26]. There are pioneering papers that focus on neuroimaging and apply this technique, based on brain MRI data.

For instance, in the study of [27], a novel approach was proposed for learning the manifold of brain MRIs. The authors proved that the learned manifold coordinated capturing shape variations of the brain. Moreover, a deep learning-based method was used to discover the patterns of similarity and variability within the images. The proposed learning algorithm was much more efficient than the traditional methods, and GPU-based parallelization made this training technique on 3D MRIs very practical. Another study by [28] dealt with the validation and the feasibility of their deep learning approach for functional and structural MRIs. The authors also described a novel constraint-based technique for visualizing the high-dimensional data. Based on their results, the deep learning-based methods can detect latent relations in neuroimaging data.

An interesting study by [29] dealt with the computer-aided diagnosis of Alzheimer’s Disease (AD) and Mild Cognitive Impairment (MCI) with a deep learning-based feature representation. The authors built a robust model for AD and MCI classification with high diagnostic accuracy. Using their dataset, the experiments were conducted, and the highest accuracy scores were achieved. The authors in this study [30] focused on the problems of feature representation and multimodal fusion of MRI and PET images. They proposed a deep learning method for a high-level latent and shared feature representation from neuroimaging modalities. It was indicated that the suggested approach could hierarchically identify the intricate latent patterns present in both MRI and PET by visually inspecting the trained model.

### 2.2. Object or Lesion Detection

One of the most difficult tasks for physicians is detecting objects of interest or lesions in medical images, which is a crucial aspect of medical diagnosis. The tasks usually include locating and identifying tiny lesions throughout the whole image. Computer-aided detection systems that automatically identify lesions in order to increase detection accuracy and reduce the amount of time that human specialists must spend reading them have a long history of study [26]. For example, the first CNN-based object detection system was developed as early of 1995. It used a CNN with four layers to detect nodules in X-ray images [31].

The differences between object detection and object classification are notable in a few areas. One important observation is that in a training context, the class balance is usually heavily biased towards the non-object class because every pixel is classified. Also, the difficulties in applying deep learning algorithms in a meaningful way to object detection are much the same as those in object classification. Few publications specifically address problems related to object detection, such as efficient pixel and voxel-wise image processing or class imbalance and hard-negative mining [26].

### 2.3. Image Segmentation

Medical image segmentation of organs and other substructures enables quantitative examination of clinical factors pertaining to volume and form, such as brain or cardiac analysis. Moreover, it frequently serves as a crucial first stage in pipelines for computer-aided detection. Finding the collection of voxels that comprise the interior or contour of the object(s) of interest is the standard definition of segmentation. The most often discussed topic in studies utilizing deep learning in medical imaging is segmentation. On the other hand, applying deep learning methods to the problem of lesion segmentation combines the difficulties of organ and substructure segmentation and object detection [26].

The most popular work in medical image segmentation with U-net belongs to the work by [32]. Also, in this study, using a GPU, the segmentation of a 512 × 512 image took less than a second. An extended study of [32] by [33] introduced a network for volumetric segmentation, which learned from sparsely annotated volumetric images. The proposed network extended the previous U-net architecture by replacing all 2D operations with the 3D counterparts. The authors ran 70,000 training iterations with a GPU for three days. This study could be applicable to other biomedical volumetric segmentation tasks.

In summary, deep learning approaches have become increasingly prevalent in the field of medical imaging segmentation. The particular architectures have been developed with the segmentation task as their primary focus. They have produced encouraging results that both match and frequently surpass those with fully convolutional network.

### 2.4. Registration

A typical image analysis problem is registration, or spatial alignment, of medical images, which involves calculating a coordinate transform from one medical image to another. This is frequently performed in an iterative framework where an assumed, particular kind of (non-)parametric transformation is carried out, and optimization is performed for a pre-established metric. Researchers have shown that deep networks can help achieve the greatest potential registration performance, despite the fact that segmentation and lesion detection are more prominent applications in medical image analysis [26]. There are some papers in the current literature that have tried to optimize registration algorithms, such as [34,35,36].

### 2.5. Content-Based Image Retrieval

Identifying related case histories, comprehending unusual conditions, and eventually enhancing patient treatment are all possible with content-based image retrieval (CBIR), a method for knowledge discovery in large databases. The primary obstacle in the advancement of CBIR techniques is the extraction of efficient feature representations from the pixel-by-pixel data and their correlation with significant ideas. Presently, some methods extract feature descriptors from medical images using (pre-trained) CNNs such as [37].

## 3. Literature Review

The primary question that establishes a framework for the current research on GPU-based parallel processing techniques for brain MRI manipulations in the WoS between 2019 and 2023 is addressed in this review: Which GPU-based parallel processing techniques, when combined with appropriate algorithms, allow for faster computation when analyzing brain MRIs? This question primarily directs our investigation into particular techniques and procedures that may be applied during the brain MRI analysis phase. Furthermore, the following additional questions are considered:

- Which medical image processing and imaging tasks are considered?
- Which methods are considered for analyzing brain MRIs?
- Which MRI sequences are involved?

The ultimate goals of this review basically are to research and evaluate the most sophisticated computer algorithms and computational techniques that allow for quick computation for medical-related tasks, particularly brain MRI investigations.

### 3.1. Methodology of Review

While conducting a keyword-based search from the WoS, some previously published works on GPU-based parallel processing for brain MRIs have higher priority than other works. Therefore, both inclusion and exclusion criteria for this review are shown in Table 1. We first conducted a keyword-based search based on the keywords “GPU-based parallel processing” and “brain” and “MRI”, then according to Table 1, we filtered the articles between 2019 and 2023 using both inclusion and exclusion criteria. For instance, the article types we used for the inclusion criteria are experimental studies and theoretical studies, published as research papers. On the other hand, we did not include in-print papers, accepted papers, conference papers, editorial texts, brief reports, theses, and books in this extended review study. Accessibility is the one of the most important criteria for inclusion, since we only included the above-mentioned articles that are electronically free to access. As a result, 14 articles were included in the literature matrix, as seen in Table 2. Recently, there has been an increase in the popularity of GPU-based parallel processing for MRI due to technological advancements that allow for faster computation with sophisticated algorithms concerning clinical aspects. Even though this review concentrates on some of the most well-known examples from the existing literature, research papers on brain MRI and GPU-based parallel processing are relatively rare in the last five years.

**Figure 2:**
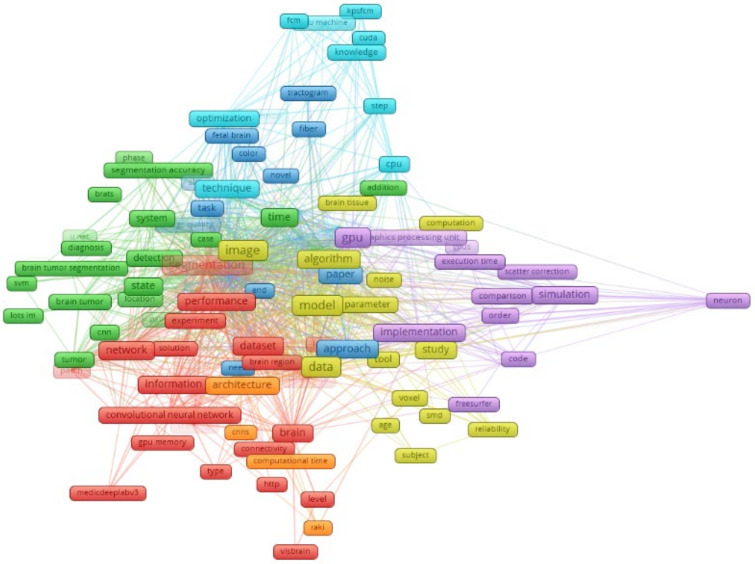
Network visualization of WOS data by searching “GPU for brain MRI” between 2019 and 2023.

The VOSviewer software (version 1.6.20) [38,39,40,41] is used to create bibliographic connection between word clouds on GPU-based parallel processing for brain MRIs, as seen in Figure 2. Therefore, in Figure 2, there are a total of seven different clusters with different colors and 122 keywords shown for the timeline between 2019 and 2023. For instance, the occurrence of the keyword “GPU” is 61, and it has 113 links to other keywords in the figure. On the other hand, the occurrence of the keyword “segmentation” is 67, and it has 107 links to other keywords.

**Table 2:** Previously published articles on GPU-based parallel processing for the analysis of brain MRIs.

Ref.	Year	Method	MRI Sequence	Task	Processing Result	Real-Time Processing
[42]	2023	Deep learning	T2	Pulse sequence simulation	High accuracy simulation	No
[43]	2023	PSO Algorithm	FLAIR, T1, T1c, T2	Segmentation	Very fast execution time	Yes
[44]	2022	G-RMOS	T1	Surface mapping	Better computation time	No
[45]	2022	MCMC	T1	Reconstruction	GPU process is faster	No
[46]	2021	SENSE	T1 and T2	Reconstruction	Reduced reconstruction time	No
[47]	2021	SVR method	T2	Reconstruction	90 times faster GPU	No
[48]	2021	Monte Carlo	T2	Simulation of a human head	Further speed by GPU	No
[49]	2020	Fuzzy C-Means	T1	Segmentation	7 times reduced segmentation time	No
[50]	2020	FreeSurfer	T1	Brain structure models	70% reduced analysis time	No
[51]	2019	Artificial Neural Network	T1	Bilateral filtering	A speed-up factor of 208 for GPU	No
[52]	2019	Artificial Neural Network	T1	Image restoration	Reduced execution time with GPU	No
[53]	2019	Microstructure estimation	T1	dMRI computation	Better performance than 200 CPU	No
[54]	2019	GLRLM Algorithm	T1 and T2	Feature extraction	Over 5 fold increase in speed	No
[55]	2019	BET Algorithm	T1	Brain extraction, visualization	1–2 s brain visualization	No

### 3.2. Results of Review

We basically examine key research publications about the integration of various high-performance computing technologies with medical image processing and analysis approaches in Table 2. For instance, in the study by [42], the authors aimed to develop a deep learning-based simulation model called Simu-Net to speed up the process of Bloch simulation. In comparison with GPU-based MRI simulation software, the simulations in this study were successfully accelerated by Simu-Net. According to the authors, Simu-Net has the potential for rapid approximation of the physical process of MRI and the optimization of MRI pulse sequences. The results of the simulation also showed the flexibility, reliability, and efficiency of the proposed framework. Another study by [43] aimed to propose an automated hardware architecture for the segmentation of MRI images in order to show differences in brain tissues. For this purpose, the authors used the Particle Swarm Optimization (PSO) algorithm in software GPU for improvements in velocity and position as well as fitness function. Basically, the goal was to create a real-time automatic system for MRI segmentation with metrics such as dice score and specificity. The proposed system achieved very high accuracy for the segmentation of MRIs from the BraTS 2013 dataset.

A study by [44] proposed G-RMOS (GPU-accelerated Riemannian metrics on surface), which included three GPU kernels: (1) GPU computing capability with a batch scheme, (2) caching in the GPU block, and (3) GPU cycle using parallelism. G-RMOS achieved important acceleration in surface mapping and used less memory than RMOS. Also, since it controlled hardware resource demands via a sub-batch scheme, the proposed framework in this study is adaptable to a wide range of system environments. In the study by [45], the authors compared the CPU and GPU bedpostx outputs through a number of trials of these algorithms on the same brain diffusion data using Markov Chain Monte Carlo (MCMC). The results showed few statistically differences between CPU and GPU bedpostx algorithms in shape localization throughout the whole brain. Despite small differences between them, the results are comparable due to the operation order and library usage.

A study by [46] proposed a calibrationless Compressed Sensing (CS) regularized by SENSE (SENSitivity Encoding) pMRI for providing MRI images with accurate clinical information. According to the simulation results, the proposed method enabled an important visual quality improvement on the MRIs in comparison with methods in the literature. Moreover, the proposed method provided faster reconstruction times compared to methods proposed in the literature. According to [47], a slice-to-volume reconstruction (SVR) method was proposed to deal with motion artifacts and provide high-quality 3D image data of brain MRIs. The results showed that the computational performance of the proposed GPU-based framework was 90 times faster than traditional CPU methods. One of the most important points of this study was that the kernel window size has a direct relation to the running time, which means a larger window size necessitates a longer processing time.

In the study by [48], a massive and parallel, multi-purpose Monte Carlo code was developed for the simulation of complicated structures, which are modeled with MRI images. The code was specifically created to run in parallel on a cluster of Linux-based computers that have several GPUs due to NVidia CUDA technology. The simulations used in this study required 2 GB of GPU memory, which was used for a human head model. The proposed code provided high-resolution and time-resolved simulations. Another study by [49] used Fuzzy C-Means (FCM) for the tissue segmentation of human brain MRI, using this method on both CPU and GPU. Based on the results of this study, the proposed parallel FCM implemented on GPU accelerated medical image segmentation when compared to the traditional CPU implementation. Also, the overall system is an example of a knowledge-based system for improving the performance of the FCM algorithm for brain tissue segmentation.

Based on the study by [50], the authors tried to accelerate the analysis time of their tool, FreeSurfer, to create models of brain structures using GPU and CUDA. FreeSurfer is a tool that takes any T1-weighted MR image as input. According to their results, analysis time was reduced by 70% using GPU acceleration compared to CPU acceleration. In the study by [51], a GPU-based bilateral filtering scheme using an efficient Artificial Neural Network (ANN) model was developed, since the application area for brain MRI restoration is very limited. The CUDA architecture was used to speed up the process of bilateral filtering computation. According to what the authors proposed for future research, the acceleration of 3D image filtering is worthy of investigation in order to create cleaner images.

The study by [52] proposed an automatic noise removal system depending on accelerated collateral filtering for brain MRIs. The proposed system used parallel computing using GPU architecture. In this study, optimal filter parameters were selected, and automation was actualized using ANN. According to the experimental results, the proposed image restoration algorithm performed faster than traditional collateral filtering, achieving noise reduction for brain MRI images. In the study by [53], two different methods for speeding up the process of diffusion MRI (dMRI) computations using GPU were presented. The first method focused on performing biophysical modeling and microstructure estimation, and the second method focused on performing tractography and long-range connectivity estimation. As a summary of this study, the authors showed that well-designed GPU-based systems could provide the same performance as hundreds of CPU cores.

In the study by [54], a new paradigm based on mature parallel primitives was proposed in order to generate gray level run length matrix (GLRLM) in a single image. Also, in order to speed up the process, GPU computing technology was used. Based on the experiments conducted in this study, this paradigm was easy to apply and offered acceleration that sped up the whole procedure. According to the study by [55], an end-to-end fast brain extraction and visualization were proposed using the brain extraction tool (BET). All of the operations in this study were performed by modern OpenGL pipelines running on a GPU. The method that the authors of this study proposed achieved a high mean Dice coefficient and mean time cost in comparison with all of the other methods from the literature.

The selected papers between 2019 and 2023 explain how medical image processing and analysis activities can be supported by high-performance computing systems, such as multi-core and GPU. The scientific community is able to make significant advances in medicine by combining parallel computer solutions with medical image processing and analysis algorithms. This review will also be helpful in the development of new research that assesses and contrasts various medical image processing and analysis techniques that are powered by high-performance computer systems.

## 4. Discussion

A comprehensive and updated overview of medical image processing and analysis methods that have been put into practice using high-performance computing technologies is provided in this review. The processing time of methods for processing and analyzing medical images has been significantly decreased by the use of high-performance computing techniques, which has made these methods appropriate for routine clinical use. In summary, these methods were employed to fully utilize the processing capacity that is frequently accessible in contemporary high-computing architectures, including multi-core and GPU.

In addition, according to the study by [56], GPUs provide powerful floating-point operations and high memory bandwidth in terms of data parallelism compared to Intel CPUs over the years. Normally, GPU can only be used for graphics rendering. The Compute Unified Device Architecture (CUDA), a parallel computing platform and programming paradigm for GPUs that simplifies programming and enables developers to utilize C/C++ as a high-level language, was launched by NVIDIA in 2007. The widespread usage of GPUs in general-purpose computing began with the introduction of the CUDA programming paradigm. Scientific computing applications including molecular dynamics (MD), CFD, weather forecasting (WF), direct simulation Monte Carlo (DSMC), medicine, and artificial intelligence already make extensive use of GPUs.

**Figure 3:**
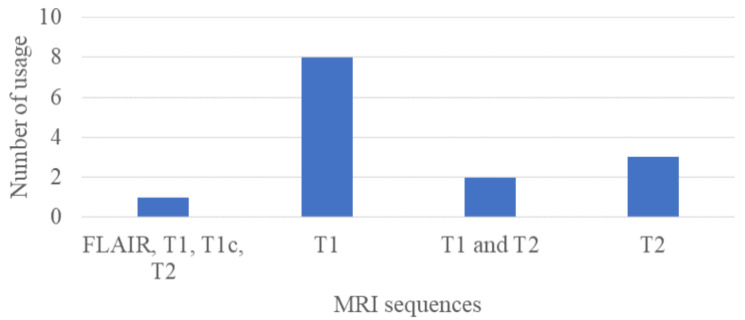
Number of MRI sequence types used in previous studies.

In Figure 3, we show the number of MRI sequences used in the previous studies from Table 2. According to this figure, the most used MRI sequence type is T1-weighted, and the sequence was used for different purposes such as segmentation [43] in 2023, image restoration in 2019 [52], reconstruction both in 2021 and 2022 [45,46], surface mapping in 2022 [44], and feature extraction in 2019 [54]. The second most used MRI sequence type is T2-weighted, and it was used for various tasks such as pulse sequence simulation in 2023 [42], reconstruction in 2021 [47], and simulation of a human head in 2021 [48]. The T1-contrast and the FLAIR sequences were used for the purpose of segmentation.

**Figure 4:**
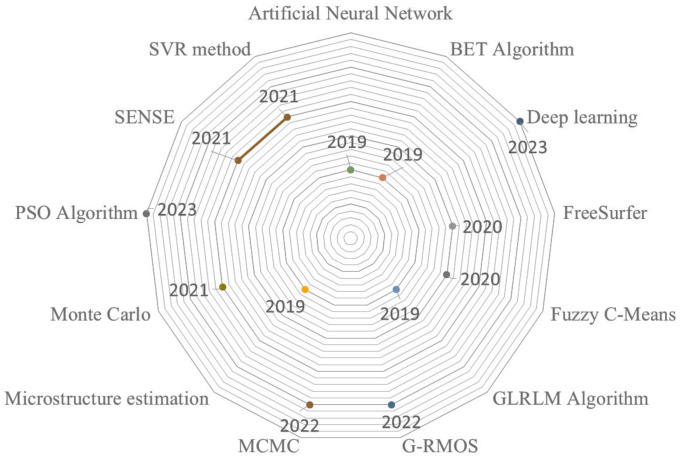
Usage of methods over the years.

In Figure 4, we also show the usage of different methods for each year between 2019 and 2023. These methods were generally combined with GPU-based parallel processing for the analysis of brain MRIs. As seen from Figure 4, there are various methods and algorithms for analyzing brain MRIs for any purposes such as segmentation, reconstruction, and simulation of brain structure models.

At present, GPU computing power has surpassed CPU processing capability. Additionally, the memory chips and CPU cores of multi-processor GPUs of today are segregated. Because the NVIDIA GTX 680, for example, has eight multi-processors with 192 processor cores in total, calculations are carried out by conforming a large number of threads, each of which is separated into an individual thread block that is under the supervision of a different multi-processing unit. Therefore, the capacity of the main method to be parallelized greatly influences the speed of contemporary GPU implementations. Algorithms for image processing are typically parallel by nature; thus, it is usually not too difficult to modify them to work on GPUs. For example, GPU parallelism is well-suited for image registration algorithms, since the GPU can calculate any desired degree of similarity in parallel, but the CPU can only perform serial optimization [9].

This study intends to provide an updated systematic literature assessment of the field, even though the number of researchers integrating high-performance computing and medical image processing and analysis approaches has seldom risen in recent years. The scientific publications that are chosen for this review study provide useful information to researchers working in these two domains. In particular, the articles discuss approaches, strategies, MRI sequences, and the tasks that are most commonly addressed. For those who plan to develop, assess, and compare algorithms used in medical image processing and analysis accelerated by high-performance computing architectures, this will be extremely valuable as it lays out the contributions made by each selected article and identifies remaining research gaps.

## 5. Conclusions

In light of the rapidly expanding applications of parallel processing in medicine, parallel computers should be employed more frequently for brain MRIs. Thus, this review emphasizes the importance of GPU-based parallel processing tools for brain MRI analysis with various medical image analysis tasks. Following a systematic compilation and presentation of recent developments that takes into consideration new perspectives, the following sentences represent the summary of achievable future prospects:

- Even though the supercomputers that are currently in use are quite powerful, more research should be conducted in order to build faster supercomputers in the future.
- Supercomputers are useful in computer science fields like quantum mechanics, weather forecasting, and molecular modeling, but more should be done to apply them in the field of medicine.
- It is possible to design special-purpose supercomputers specifically for use in medical applications; some are even devoted to astrophysics and even chess playing.
- Grid computing should be integrated with supercomputers to handle more complex medical computing tasks, such as brain MRI analysis.
- In medicine-related research, the capabilities of current supercomputers should be evaluated and compared for the event of an emergency.
- Applications for mobile phones that are dependable, fast, strong, parallel, user-friendly, affordable, and intelligent should be created for doctors to aid in the treatment of neurodegeneration, which is a major burden that increases geometrically with the number of patients, costs of care, and drug therapy.
- To solve complex molecular processes and new drug interactions with human tissues, more supercomputers should be built for the purpose of designing new medications.

We explore the potential benefits of GPU programming and parallel processing for brain MRI image processing. Additionally, we illustrate the key distinctions between CPU and GPU programming as well as their underlying general ideas. Based on their unique application areas and implementation on human brain MRI, relevant works on parallel processing for brain MRI analysis have been carefully selected from the WOS. We compile a comprehensive literature matrix with a list of the chosen articles after evaluating the search results. We conduct a thorough discussion of our findings and present the future trends and prospects of this field by emphasizing the future aspects of parallel processing for brain MRI, all while taking these results into account. We believe this review can offer a thorough analysis of the subject and make a significant contribution by making the existing literature easier to understand.

## Figures and Tables

**Table 1:** Inclusion and exclusion criteria for this review.

	Inclusion Criteria	Exclusion Criteria
Article Type	Experimental	In-print papers
	Theoretical	Accepted papers
	Published research articles	Conference papers
		Editorial texts
		Brief reports
		Theses
		Books
Language	Full-text English articles	Full-text any other language articles
Interdisciplinarity	Articles from different journals potentially very relevant to the topic	Articles with certain keywords in other disciplines
Accessibility	Articles and papers that are free to access	Articles and papers that are not free to access electronically
Publication date	All articles and publications in the mentioned databases between 2019 and 2023	Online publications after review period (after December 2023)

## Data Availability

No data were created in this review.
